# Supplementary figures and images for: Modeling geographic distribution of arbuscular mycorrhizal fungi from molecular evidence in soils of Argentinean Puna using a maximum entropy approach
Source: PeerJ. 2023 Jan 12;11:e14651. doi: 10.7717/peerj.14651 (PMC9840858; doi:10.7717/peerj.14651)

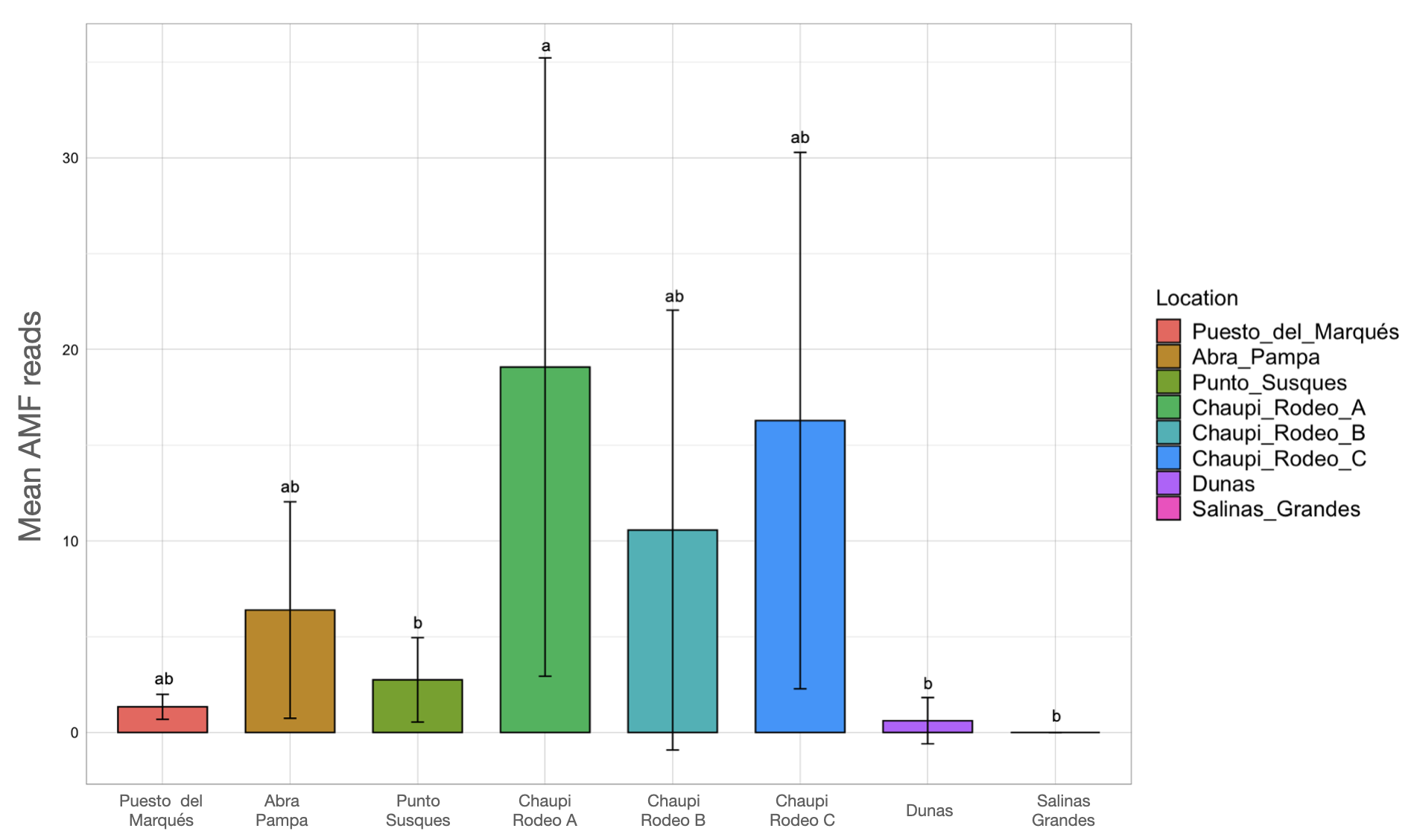

Supplement: Supplemental Information 1 — The sum of sample reads is grouped and plotted per location. Samples locations are indicated by colors as described in the legend and labelled below each bar. Different letters indicate pairwise post-hoc test statistical significances with Bonferroni correction (p <= 0.05). Note that the Salinas Grandes column color is not shown because no AMF sequences were retrieved within that location. The average number of sequences retrieved (mean AMF reads) is shown on the Y axis. [file peerj-11-14651-s001.png]

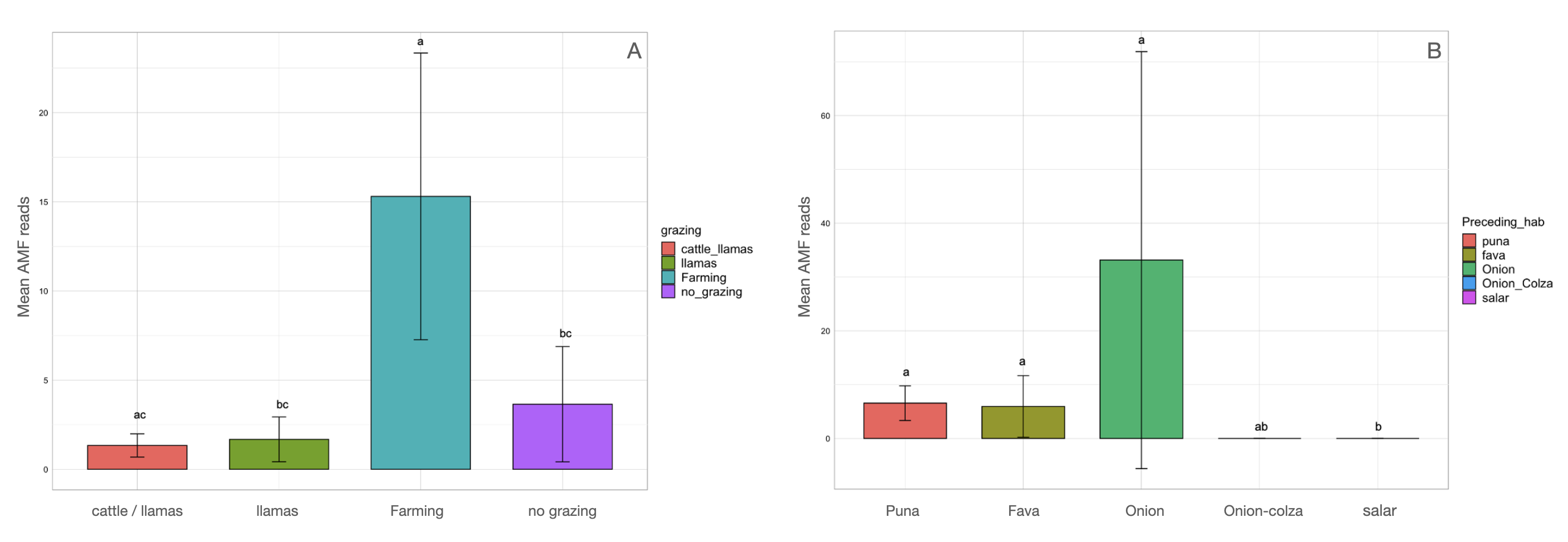

Supplement: Supplemental Information 2 — Colors refer to the contribution of the different tested groups. Group names are labelled below each bar. Different letters indicate pairwise post-hoc test statistical significance difference with Bonferroni correction (p <= 0.05). Note that some column colors are not shown because no AMF sequences were retrieved within these groups. The average number of sequences retrieved (mean AMF reads) is shown on the Y axis. [file peerj-11-14651-s002.png]

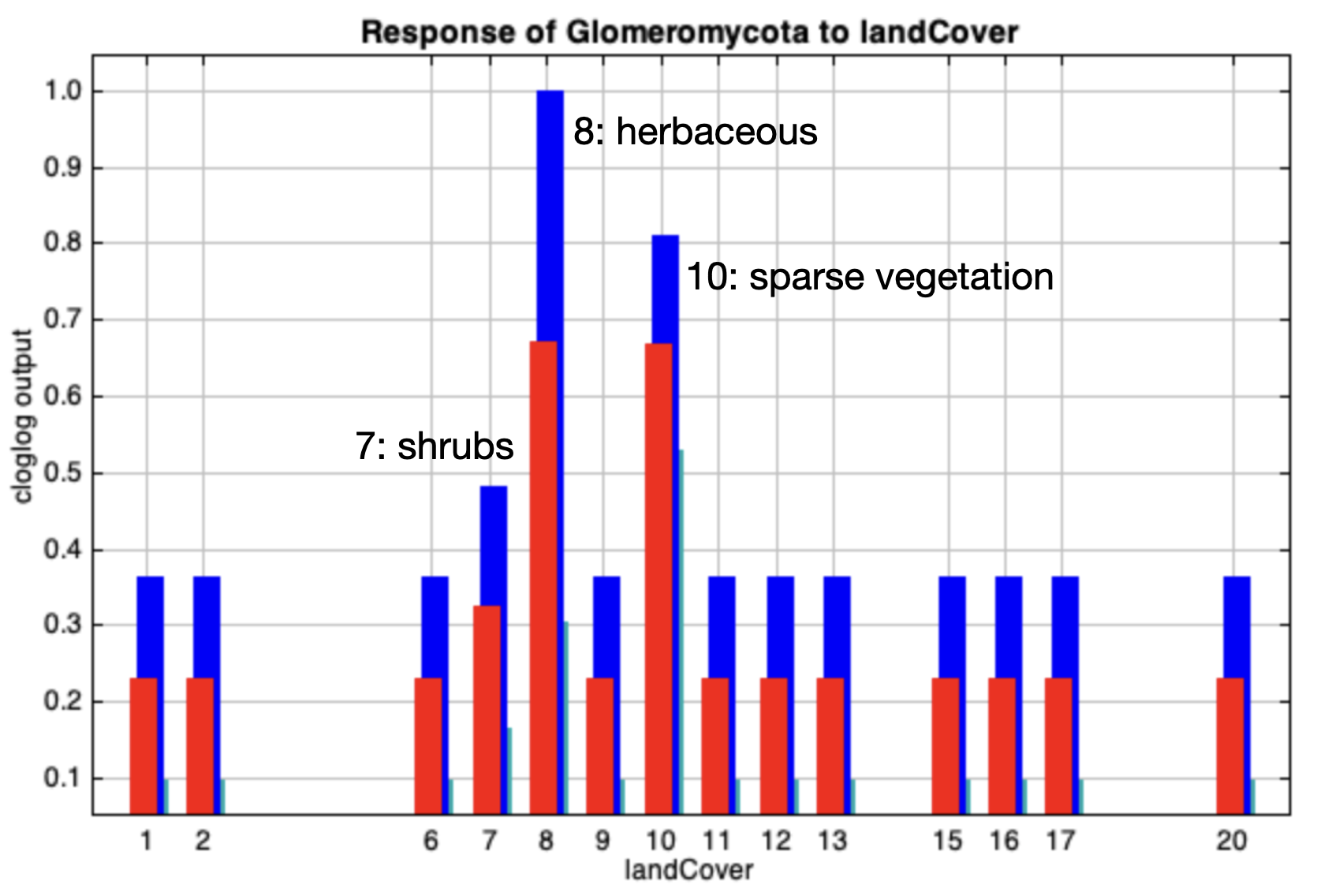

Supplement: Supplemental Information 3 — The curves show the effect of varying the chosen variable on the MaxEnt prediction, by keeping all other environmental variables at their average sample value. Labels of the highest response values are indicated. [file peerj-11-14651-s003.png]

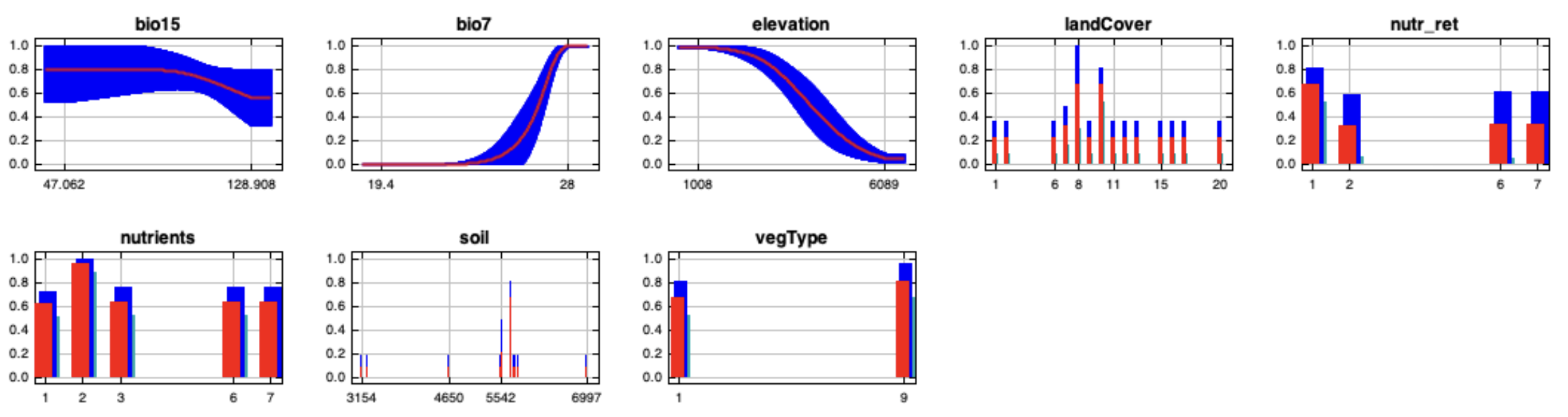

Supplement: Supplemental Information 4 — The curves show the effect of varying the chosen variable on the MaxEnt prediction, by keeping all other environmental variables at their average sample value. Worldclim bioclimatic variables Bio7 and Bio15 represent respectively the temperature annual range and the precipitation seasonality. [file peerj-11-14651-s004.png]

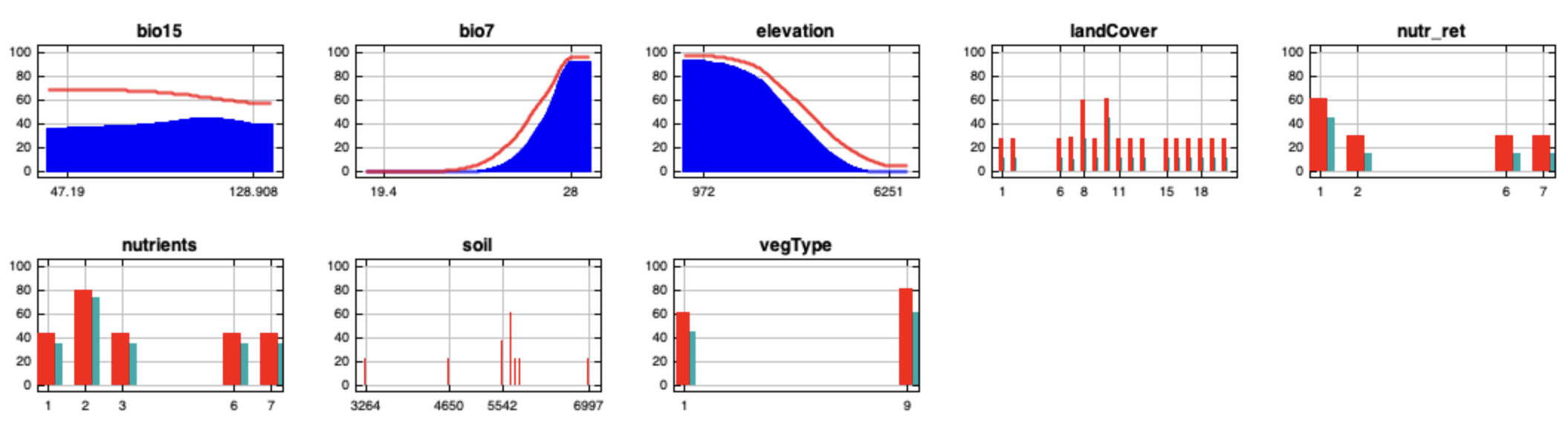

Supplement: Supplemental Information 5 — The curves show the effect of varying the chosen variable on the MaxEnt prediction, by keeping all other environmental variables at their average sample value. Worldclim bioclimatic variables Bio7 and Bio15 represent respectively the temperature annual range and the precipitation seasonality. [file peerj-11-14651-s005.png]

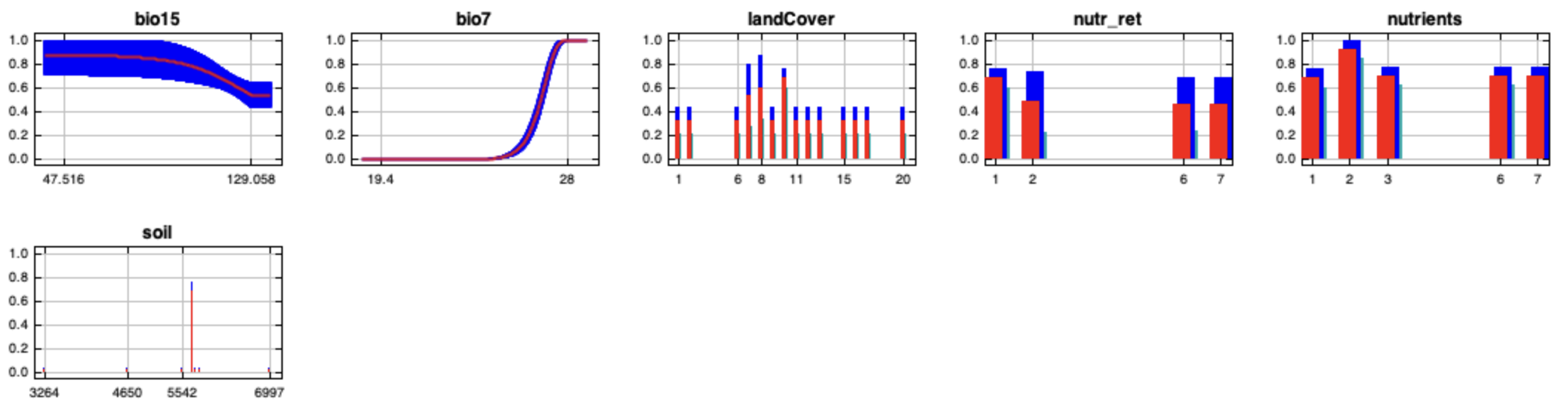

Supplement: Supplemental Information 6 — The curves show the effect of varying the chosen variable on the MaxEnt prediction, by keeping all other environmental variables at their average sample value. Worldclim bioclimatic variables Bio7 and Bio15 represent respectively the temperature annual range and the precipitation seasonality. [file peerj-11-14651-s006.png]

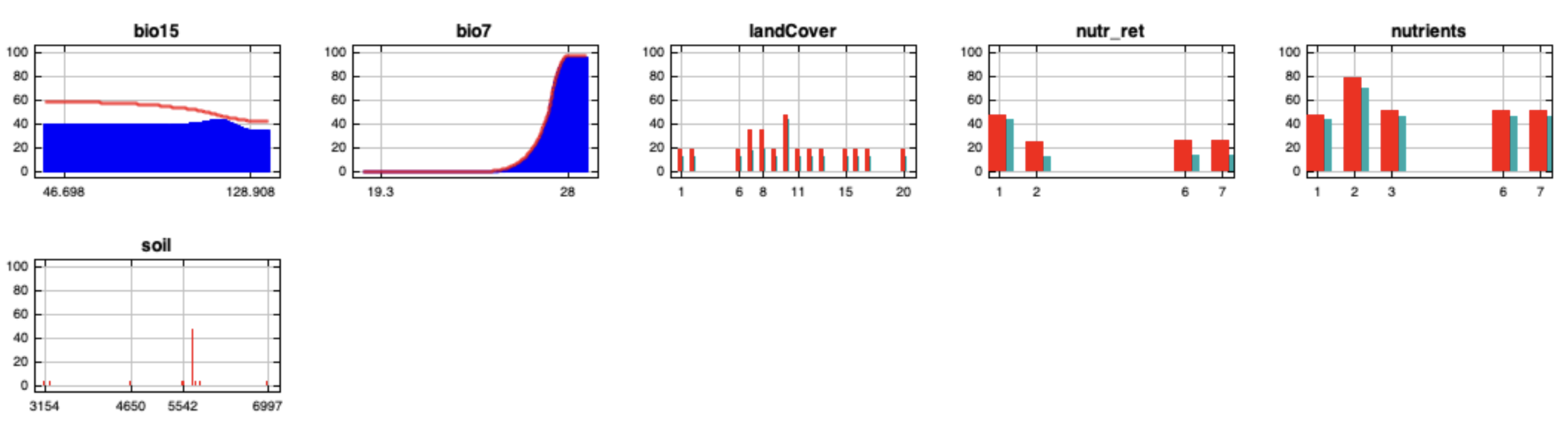

Supplement: Supplemental Information 7 — The curves show the effect of varying the chosen variable on the MaxEnt prediction, by keeping all other environmental variables at their average sample value. Worldclim bioclimatic variables Bio7 and Bio15 represent respectively the temperature annual range and the precipitation seasonality. [file peerj-11-14651-s007.png]
